# Possible effect of morphological variations of plantaris muscle tendon on harvesting at reconstruction surgery-case report

**DOI:** 10.1007/s00276-020-02463-1

**Published:** 2020-04-04

**Authors:** B. Gonera, K. Kurtys, P. Karauda, Ł. Olewnik, M. Polguj

**Affiliations:** 1 Department of Anatomical Dissection and Donation, Medical University of Lodz, Mechaniczna 5a, 92-310 Lodz, Poland; 2 Department of Normal and Clinical Anatomy, Medical University of Lodz, Lodz, Poland

**Keywords:** Achilles tendon, Anatomy, Evolution, Plantaris muscle, Plantaris tendon

## Abstract

**Purpose:**

Seemingly a well-known, weak, and vestigial plantaris muscle should not be a revelation. However, recent studies have shown that this structure is incredibly underestimated and perceived only as an infirm flexor of the talocrural joint, the knee joint or a great source of graft tissue. Usually, the origin of this inconspicuous muscle begins at the lateral supracondylar line of the femur and the knee joint capsule. It continues distally, forming a long and slender tendon. In most cases, it inserts onto the calcaneal tuberosity on the medial side of the Achilles tendon. However, many morphological variations have been discovered during anatomical dissections and surgical procedures. Nevertheless, according to the present literature, no other studies presented such a complex insertion variant, with indisputable clinical value and significant proof of development of this forgotten muscle.

**Methods:**

The dissection of the right thigh, knee, crural and talocrural region was performed using standard techniques according to a strictly specified protocol.

**Results:**

Four different insertion points were observed. The first band (A) inserted near to the tarsal canal flexor retinaculum. The second band (B) bifurcates into two branches—B1 and B2. B1 is located on the medial side and B2 is located on the lateral side of the calcaneal tuberosity. The third band (C) is inserted into the superior nonarticular calcaneal surface of the calcaneus anteriorly to the Achilles tendon.

**Conclusion:**

A differently shaped plantaris tendon could be considered a cause of harvesting procedure failure. In the light of new case reports perhaps what we are now witnessing is remodeling and transformation of the Plantaris muscle. If so, the awareness of the influence on the onset of Achilles midportion tendinopathy or a potential role in tibialis posterior conflict can be crucial for every clinician.

## Introduction

The plantaris muscle (PM) is typically described as a small, short and fusiform muscle in a part of the superficial posterior compartment of the lower limb. In most cases, PM originates from the lateral supracondylar line of the femur and the knee joint capsule. However, it may also arise from the inferior division of linea aspera; fascia covering the popliteus muscle; the fibula; the oblique line of tibia or even the soleus muscle [6, 9, 14, 17]. Being surrounded by gastrocnemius muscle, as well as soleus muscle, and having very long tendon and relatively short muscle belly, PM seems to have an insignificant influence (0.7% of power of plantar flexors of the foot) on the knee and ankle joint biomechanics [31].

Insertion as well as origin are subject to many variations [6, 34]. Generally, the PM is characterized by a wide, fan-shaped distal attachment to the calcaneal tuberosity on the medial side of the Achilles tendon. Nevertheless, according to comprehensive studies [6, 9, 26], four more types of insertion could be described: three with their insertion point at the calcaneal tuberosity and one with its insertion into the deep crural fascia. Interestingly, when the Plantaris tendon (PT) is developed independently of the Achilles tendon, it remains intact when the Achilles tendon ruptures [8].

Although PM is considered vestigial in humans, it plays an important role as an organ of proprioceptive function for larger, more powerful plantar flexors as it contains high density of muscle spindles. There are even nine times more muscle spindles per gram in the plantaris muscle than in the gastrocnemius muscle [28].

The specific course of the PT and type of insertion may significantly affect the onset of Achilles midportion tendinopathy [25]. Despite extensive research, the pathogenesis is still not fully understood [4]. Nevertheless, clinical studies have shown that removing PM may result in an improvement of the Achilles tendon structure, accompanied by improved clinical VISA-A scores [18], i.e. a quantitative index of pain and function in patients with Achilles tendinopathy.

For almost one hundred years, many surgical professionals, have found PM tendon very useful. Due to a simple harvesting technique, it is used as a potential donor of graft for various reconstructions [2, 19]. However, because of high variability, diversity or even absence of PM insertions, the harvesting procedure appears to be much more complicated than previously assumed.

The aim of this study is to present a unique and complex insertion of the plantaris muscle which has never been described before. Awareness of such diverse insertion points may be crucial while planning a tendon grafting procedure. Moreover, the author of the study also deliberates whether the PM may be still perceived as a vestigial muscle.

## Case report

**Fig. 1:**
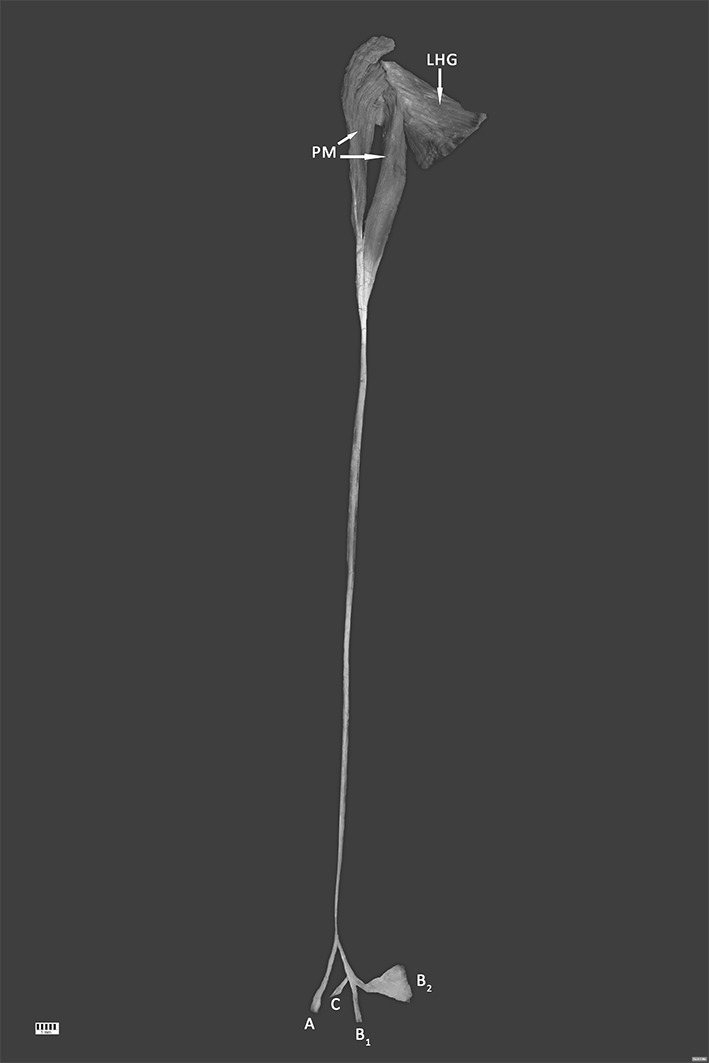
Unique plantaris muscle (PM) and tendon (PT)—isolated muscle (distal attachments are retracted for better understanding the emerging points). Four different types of insertion are presented here: A, B1, B2, C. *PM *plantaris muscle (2 heads), *LHG *lateral head of gastrocnemius

**Fig. 2:**
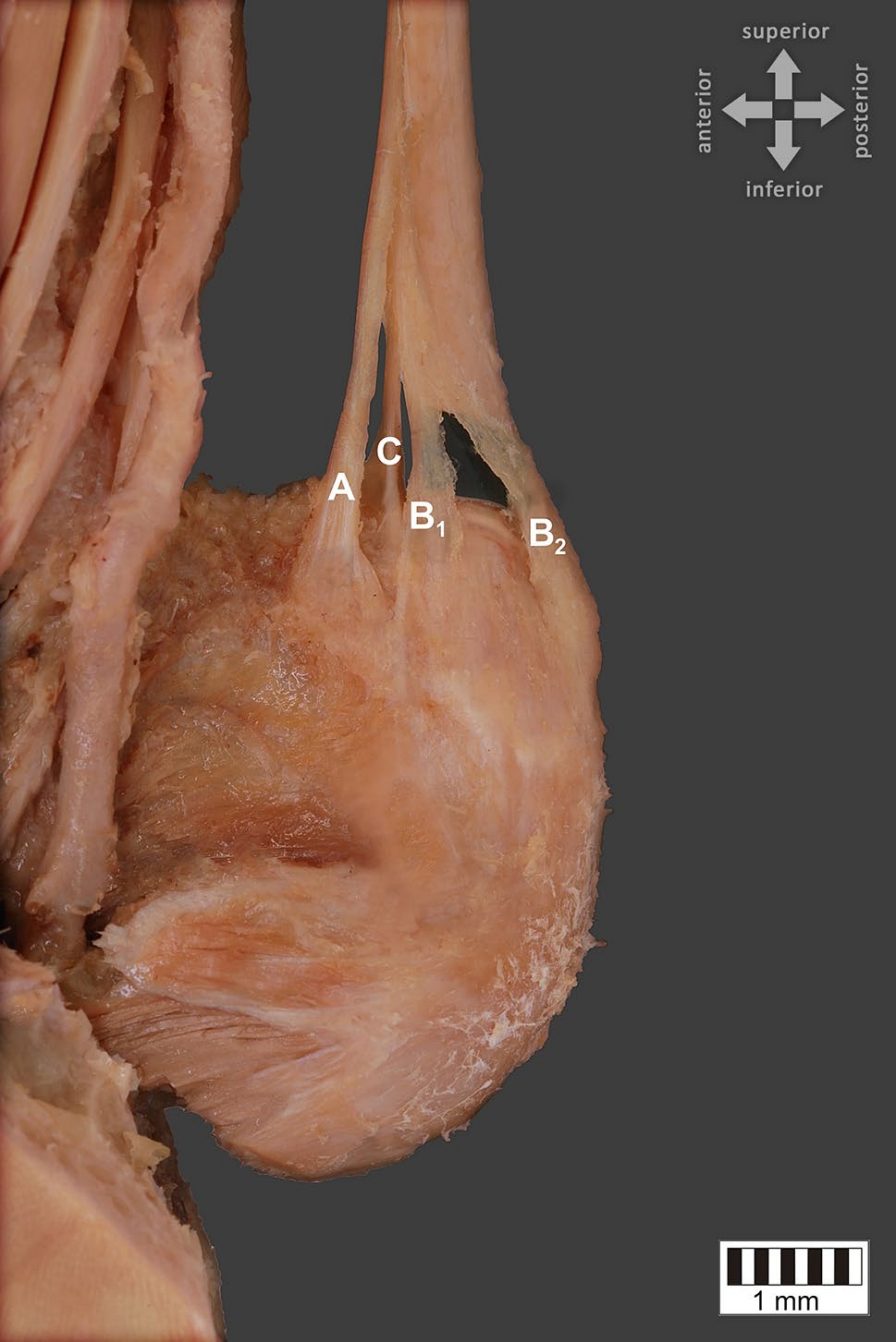
Unique plantaris tendon (PT) distal attachments. Four different types of insertion are presented here: A**—**emerged from the main PT and is inserted into the middle rough area on the posterior surface of calcaneus near to the tarsal canal flexor retinaculum, B1**—**located on the medial side of the calcaneal tuberosity, B2**—**located on the lateral side of the calcaneal tuberosity, C**—**emerged from the main PT and is inserted into the superior nonarticular calcaneal surface of the calcaneus anteriorly to the Achilles tendon. Posteromedial view

**Fig. 3:**
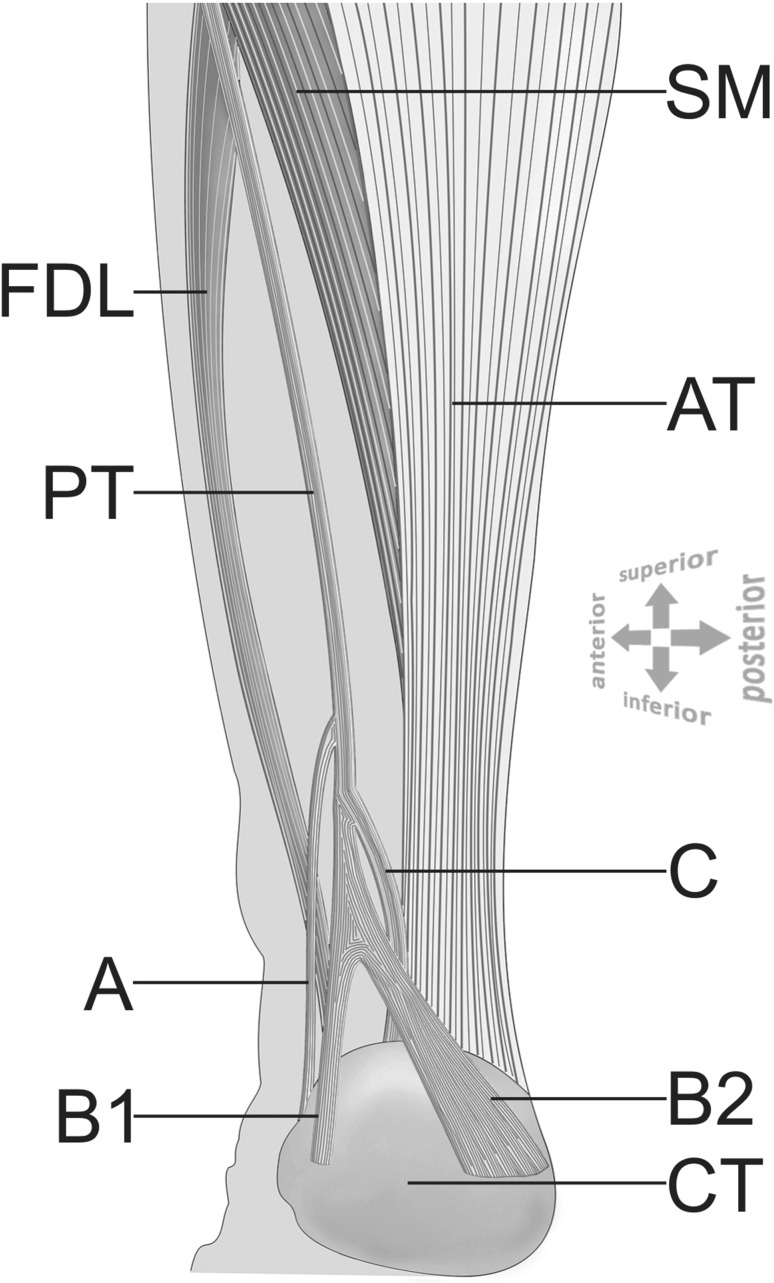
Unique plantaris tendon (PT) distal attachments—scheme. Four different types of insertion are presented here: A, B1, B2, C. *AT* Achilles (calcaneal) tendon, *SM* soleus muscle, *CT* calcaneal tuberosity, *FDL* flexor digitorum longus. Posteromedial view

**Fig. 4:**
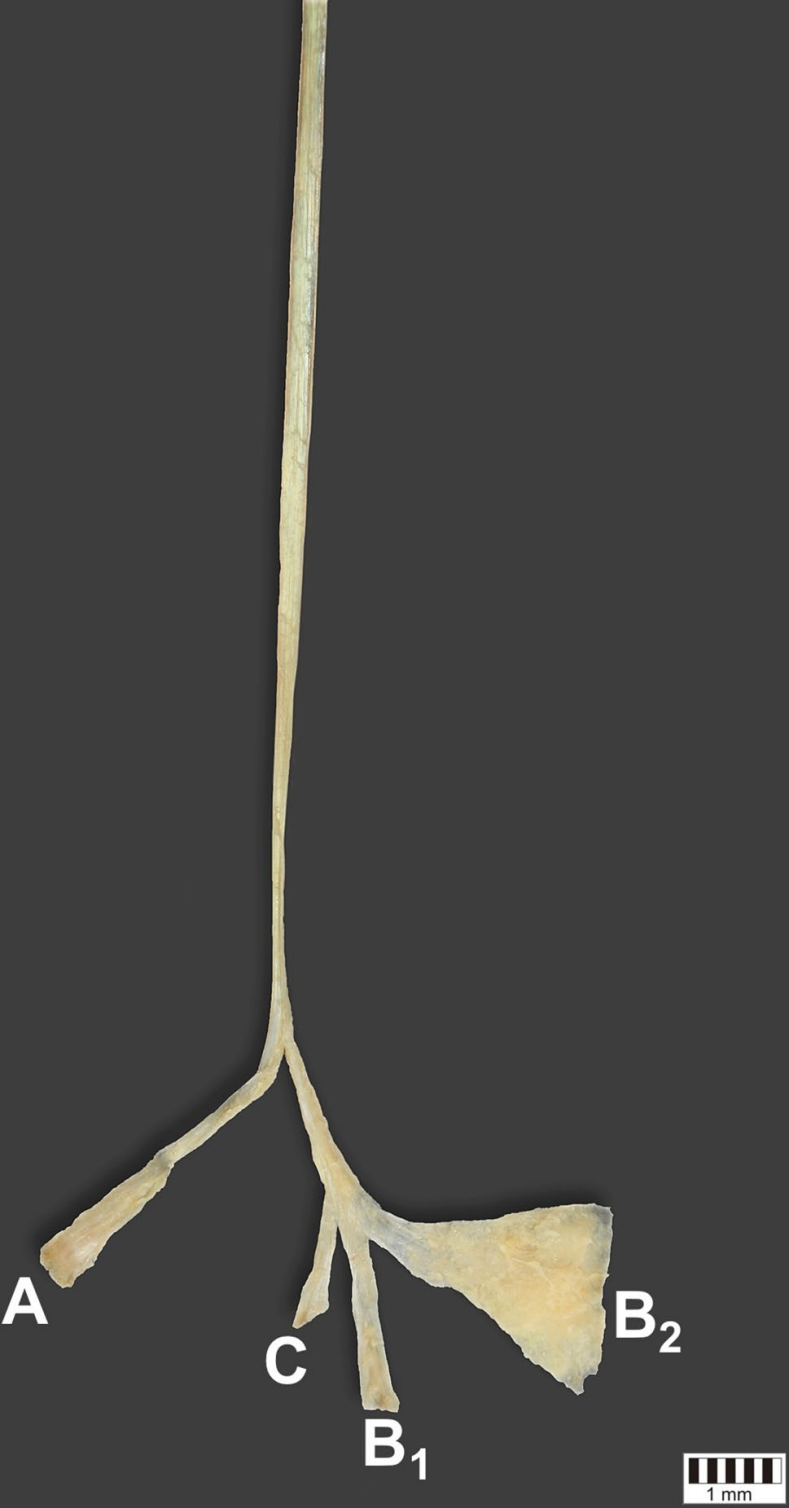
Unique plantaris tendon (PT) distal attachments—isolated tendon (distal attachments are retracted for better understanding the emerging points). Four different types of insertion are presented here: A, B1, B2, C

The dissected male cadaver, aged 68 years old at death, was subjected to a routine anatomical dissection for research and teaching purposes at the Department of Normal and Clinical Anatomy of the Medical University of Lodz. The dissection of the right thigh, knee, crural and talocrural region was performed using standard techniques according to a strictly specified protocol [22–24]. An absolutely unique insertion variant of PM was discovered during this procedure (Fig. 1). What is also interesting the PT was completely separated from the Achilles tendon at proximal (Fig. 3). It run in the space between the Gastrocnemius muscle and the Soleus muscle. Moreover being without any fascia connecting it to Achilles tendon. Not only one but 3 different bands and 4 different insertion points were observed (Figs. 2, 3, and 4):

- The first band (A) emerged from the main PT and is inserted into the middle rough area on the posterior surface of calcaneus near to the tarsal canal flexor retinaculum
- The second band (B) is the prolonged main PT which bifurcates into two branches—B1 and B2. Both are located on the calcaneal tuberosity. B1 is located on the medial side and B2 is located on the lateral side of the calcaneal tuberosity.
- The third band (C) emerged from the main PT and is inserted into the superior nonarticular calcaneal surface of the calcaneus anteriorly to the Achilles tendon

The next stage of the procedure involved gathering detailed morphometric measurements. After photographic documentation, the PT was carefully dissected to minimize a measurement mistake (Fig. 4). The measurements were taken based on digital photographic images processed through MultiScanBase 18.03 (Computer Scanning System II, Warsaw, Poland).

The measurements results were the following:

- The length of the band A was 35.86 mm. The width of the band in the widest point was 4.82 mm. The width of the band in the narrowest point was 1.70 mm
- The length of the band B1 was 21.23 mm. The width of the band in the widest point was 3.37 mm. The width of the band in the narrowest point was 2.23 mm
- The length of the band B2 was **2**8.44 mm. The width of the band in the widest point was 19.40 mm. The width of the band in the narrowest point was 3.40 mm
- The length of the band C was 14.12 mm. The width of the band in the widest point was 3.12 mm. The width of the band in the narrowest point was 1.85 mm

## Discussion

Specific location, inconstancy, variability have led to many inexact and unclear theories about evolutional development of PM. In the nineteenth century, the first research studies were conducted and strongly suggested that the plantaris muscle is a rudimentary muscle that plays a minor role in gait biomechanics. Moreover, due to bipedal posture transition, its insertion migrated from plantar aponeurosis towards the calcaneus as a result of adaptation [5, 37]. In the process of posture verticalization, the heel came in contact with the ground and the foot acquired the position of 90° in relation to the lower limb, and plantar aponeurosis gradually developed insertion on the lower side of calcaneus while its muscular part regressed, as it was emphasized by Daseler and Anson [6]. A similar disparity that supports PM vestigiality applies to the frequency of PM absence. As it is commonly known, what characterizes devolution is being highly variable in frequency within one population or between populations. Former scientific research revealed that the presence ranged from 91 [32] to 87% [10] and even up to 81.8% [13]. However, remarkably different values were shown in more recent studies—the presence values started with 96% [26] and were as high as 100% [2, 34].

Nowadays PM is considered by anatomists and embryologists a derivative of a deeper portion of the lateral head of the gastrocnemius and is often called “gastrocnemius tertius”, which represents the third head of gastrocnemius due to its origin, which is often connected to the lateral head of gastrocnemius [20]. More interestingly, researchers focused on PM innervations in contrast to anatomists who showed that this relationship seems to be much more complicated and reconsideration may be needed [21]. The nerve trunks to the PM arose from the nerve group of the deep posterior crural muscles rather than from the nerve groups of the superficial crural muscles and thus PM may be derived from anlage of the deep posterior crural muscles [21]. That clearly indicates that the perception of PM has changed over decades and our paper reveals how much we still do not know and how much could be discovered in future research about the structure that may be involved in the remodeling process instead of devolution as it was previously thought.

As it is mentioned above, the plantaris tendon is often used as a potential donor of a graft in various reconstructions. According to the studies of Aragao et al. [2], PT was harvested for flexor tendon replacement in hand surgery, as well as lateral ankle ligaments. Furthermore, it is often used in tendon reconstruction of the hand when the palmaris longus is absent [35] (which is reported to be absent more often than PT [36]) and indications for its use are similar to those in the event of palmaris longus [38]. The palmaris longus muscle is said to be homologous with the plantaris muscle and that they are equivalent to each other [12]. However, Vanderhooft [36]. claimed that there is no significant correlation between the palmaris longus and plantaris muscles and thus it could be speculated that PM in contrast to palmaris longus is still developing. An extremely tensile structure of PT was also appreciated for atrioventricular valve repair [30] where it was speculated that the replacement of the papillary muscle, chordae, or both with Plantaris tendon could be performed. It is of clinical significance that in contrast to palmaris longus tendon which may be detected clinically, the PT is not amenable to palpation. In cases where PT is considered a graft source, ultrasound [32] and MRI [29] performed preoperatively, appeared to be very helpful in confirming that the muscle will make a good graft [39].Well-performed ultrasound and MRI procedures may be crucial to avoid mistakes and may help improve the planning of surgical procedures, because every uncommon formation of PT, such as the one presented in this study, may increase the risk of failure while harvesting the tendon. Differently shaped tendons can affect the ease of tendon harvesting.

Nowadays, harvesting the plantaris tendon is performed in accordance to McCarthy JG [19] method which suggests that a 2–3 cm incision be made anteriorly to the Achilles tendon on the medial aspect of the ankle, 1 cm proximally to the most proximal part of the calcaneus. The plantaris tendon, which usually lies close to the medial aspect of the calcaneus, is divided. A Brand tendon stripper is slipped over the free end of the tendon and advanced under tension for approximately 20 to 25 cm, until the cutting edge strikes the belly of the plantaris muscle. Hypothetically, due to application of this standard dorsomedial method, PT may remain unnoticed when the tendon is anterior to the Achilles tendon, which Olewnik et al. [25] distinguished as variant B of the course of the PT in the most recent PT course and insertion classification. According to their work, this variant is present in 15.5% of all specimens. However, as it is mentioned above, more complications may occur while harvesting the plantaris tendon. We would like to attract surgeons’ attention to the fact that in cases similar to the one presented in our work, it is highly probable to tear the tendon if it is bifurcated. Therefore, we would like to extend the existing classification by adding a new type, called “rare cases”. Apart from all previously discovered case reports, every newly reported PT with accessory bands will be included. Considering Plantaris muscle a structure in a remodeling process, a much greater number of insertion variants could appear in the nearest future. Such classification could shed new light on a complex problem of harvesting Plantaris tendon. Possibly a new harvesting method, which includes bypassing emerging points, may be needed.

An increasing number of Achilles tendon disorders, including tendinopathy, is being recorded [1, 3, 7, 15, 16]. Potential involvement of PM in midportion Achilles tendinopathy, which accounts for 55–65% of cases, has been described in numerous research studies which are growing in number [1, 33, 34]. According to the studies of Olewnik et al. [25], one PT type, in particular, should be taken into consideration, i.e. insertion into the flexor retinaculum of the leg. It is so remarkable because in our paper the same type is presented (band A). Interestingly, this insertion type has not been discovered before. Moreover, this area is susceptible to tendinopathy and dislocation of the tibialis posterior muscle [11, 27]. In this type of insertion, clinicians should be aware of potential risk of developing tendinopathy or dislocation of the tibialis posterior tendon.

Although this study presents an extensively analyzed and highly attractive anatomical case, it has a few limitations. There are only assumptions and speculations based on previous studies that it may cause a failure of the harvesting procedure. Further and more clinical researches should be conducted to identify more such cases which should be next examined to point out problems encountered while using tendon striper. Nevertheless, the aim of our study is to raise awareness of such possibility and this may be the first step to solve this problem.

## Conclusion

This case study presents an extremely unique and complex insertion of the plantaris muscle. An undeniable influence of such ramified tendon on harvesting procedure is proven. A differently shaped plantaris tendon could be considered a cause of its failure. In the light of new case reports which are constantly being published, a question arises: Are we now witnessing remodeling and transformation of the Plantaris muscle? If so, the awareness of the influence on the onset of Achilles midportion tendinopathy or a potential role in tibialis posterior conflict can be crucial for every clinician.

## References

[1] Alfredson H, Spang C (2018). Clinical presentation and surgical management of chronic Achilles tendon disorders—a retrospective observation on a set of consecutive patients being operated by the same orthopedic surgeon. Foot Ankle Surg.

[2] Aragão JA, Reis FP, Guerra DR, Cabral RH (2010). Presencia de Músculos Plantares y su Relación Musculotendinosa en Cadáveres Humanos Adultos. Int J Morphol.

[3] Asplund CA, Best TM (2013). Achilles tendon disorders. BMJ.

[4] Cook JL, Purdam CR (2009). Is tendon pathology a continuum? A pathology model to explain the clinical presentation of load-induced tendinopathy. Br J Sports Med.

[5] Cruveilhier J (1851). Traité d’Anatomie descriptive.

[6] Daseler EH, Anson BJ (1943). The plantaris muscle: an anatomical study of 750 specimens. J Bone Jt Surg.

[7] DeJonge S, Van Den Berg C, De Vos RJ, Van Der Heide HJL, Weir A, Verhaar JAN, Bierma-Zeinstra SMA, Tol JL (2011). Incidence of midportion Achilles tendinopathy in the general population. Br J Sports Med.

[8] Delgado GJ, Chung CB, Lektrakul N, Azocar P, Botte MJ, Coria D, Bosch E, Resnick D (2002). Tennis leg: clinical US study of 141 patients and anatomic investigation of four cadavers with MR imaging and US. Radiology.

[9] DosSantos MA, Dos Bertelli JA, Kechele PR, Duarte H (2009). Anatomical study of the plantaris tendon: reliability as a tendo-osseous graft. Surg Radiol Anat.

[10] Freeman AJ, Jacobson NA, Fogg QA (2008). Anatomical variations of the plantaris muscle and a potential role in patellofemoral pain syndrome. Clin Anat.

[11] Goucher NR, Coughlin MJ, Kristensen RM (2006). Dislocation of the posterior tibial tendon: a literature review and presentation of two cases. Iowa Orthop J.

[12] Gray H, Williams PL, Peter L, Bannister LH (1995). Gray’s anatomy: the anatomical basis of medicine and surgery.

[13] Harvey FJ, Chu G, Harvey PM (1983). Surgical availability of the plantaris tendon. J Hand Surg Am.

[14] Henle J (1866). Handbuch der systematischen Anatomie des Menschen.

[15] Järvinen TAH, Kannus P, Paavola M, Järvinen TLN, Józsa L, Järvinen M (2001). Achilles tendon injuries. Curr Opin Rheumatol.

[16] Kvist M (1994). achilles tendon injuries in athletes. Sport Med An Int J Appl Med Sci Sport Exerc.

[17] LeDouble A (1897). Traité des variations du système musculaire de l’homme: et de leur signification au point de vue de l’anthropologie zoologique.

[18] Masci L, Spang C, Van Schie HTM, Alfredson H (2015). Achilles tendinopathy—Do plantaris tendon removal and Achilles tendon scraping improve tendon structure? A prospective study using ultrasound tissue characterisation. BMJ Open Sport Exerc Med.

[19] McCarthy JG (1990). Plastic surgery.

[20] Menton D (2001). The plantaris and the question of vestigial muscles in man. J Creat.

[21] Okamoto K, Wakebe T, Saiki K, Tsurumoto T (2013). The nerves to the plantaris muscle and a bipennate part of the soleus muscle. Anat Sci Int.

[22] Olewnik Ł, Gonera B, Kurtys K, Podgórski M, Polguj M, Sibiński M, Topol M (2018). The anterolateral ligament of the knee: a proposed classification system. Clin Anat.

[23] Olewnik Ł, Gonera B, Kurtys K, Podgórski M, Polguj M, Topol M (2019). A proposal for a new classification of the fibular (lateral) collateral ligament based on morphological variations. Ann Anat.

[24] Olewnik Ł, Gonera B, Podgórski M, Polguj M, Jezierski H, Topol M (2019). A proposal for a new classification of pes anserinus morphology. Knee Surg Sport Traumatol Arthrosc.

[25] Olewnik L, Wysiadecki G, Podgórski M, Polguj M, Topol M (2018). The plantaris muscle tendon and its relationship with the Achilles tendinopathy. Biomed Res Int.

[26] Olewnik Ł, Wysiadecki G, Polguj M, Topol M (2017). Anatomic study suggests that the morphology of the plantaris tendon may be related to Achilles tendonitis. Surg Radiol Anat.

[27] Ortolani A, Bevoni R, Russo A, Marcacci M, Girolami M (2016). Posterior tibial tendon displacement behind the tibia and its interposition in an irreducible isolated ankle dislocation: a case report and literature review. Joints.

[28] Peck D, Buxton DF, Nitz A (1984). A comparison of spindle concentrations in large and small muscles acting in parallel combinations. J Morphol.

[29] Saxena A, Bareither D (2000). Magnetic resonance and cadaveric findings of the incidence of plantaris tendon. Foot Ankle Int.

[30] Shuhaiber JH, Shuhaiber HH (2003). Plantaris tendon graft for atrioventricular valve repair: a novel hypothetical technique. Texas Hear Inst J.

[31] Silver RL, de la Garza J, Rang M (1985). The myth of muscle balance. A study of relative strengths and excursions of normal muscles about the foot and ankle. J Bone Joint Surg Br.

[32] Simpson SL, Hertzog MS, Barja RH (1991). The plantaris tendon graft: an ultrasound study. J Hand Surg Am.

[33] Van Sterkenburg MN, Kerkhoffs GMMJ, van Dijk CN (2011). Good outcome after stripping the plantaris tendon in patients with chronic mid-portion Achilles tendinopathy. Knee Surg Sport Traumatol Arthrosc.

[34] Van Sterkenburg MN, Kerkhoffs GMMJ, Kleipool RP, Niek Van Dijk C (2011). The plantaris tendon and a potential role in mid-portion Achilles tendinopathy: an observational anatomical study. J Anat.

[35] Unglaub F, Bultmann C, Reiter A, Hahn P (2006). Two-staged reconstruction of the flexor pollicis longus tendon. J Hand Surg Am.

[36] Vanderhooft E (1996). The frequency of and relationship between the palmaris longus and plantaris tendons. Am J Orthop (Belle Mead NJ).

[37] Vlaic J, Josipovic M, Bohacek I, Jelic M (2019). The plantaris muscle: Too important to be forgotten. A review of evolution, anatomy, clinical implications and biomechanical properties. J Sports Med Phys Fitness.

[38] Yamazaki H, Kato H, Nakatsuchi Y, Murakami N, Hata Y (2006). Closed rupture of the flexor tendons of the little finger secondary to non-union of fractures of the hook of the hamate. J Hand Surg Am.

[39] Yammine K, Saghie S, Assi C (2019). A meta-analysis of the surgical availability and morphology of the Plantaris tendon. J Hand Surg Asian Pac.

